# Stressors, Barriers and Facilitators Faced by Australian Farmers When Transitioning to Retirement: A Scoping Review

**DOI:** 10.3390/ijerph20032588

**Published:** 2023-01-31

**Authors:** Chloe M. E. Fletcher, Louise Stewart, Kate M. Gunn

**Affiliations:** Department of Rural Health, Allied Health and Human Performance, University of South Australia, Adelaide, SA 5000, Australia

**Keywords:** ageing, agriculture, farming, occupational identity, retirement

## Abstract

Farms in Australia are largely family owned and managed. Complex interactions between farming history, traditions, family, business, succession, identity and place can lead to difficulties in planning for retirement for farmers. Due to the significant implications of this for farmers’ health and wellbeing, there is a clear need to determine how farmers may be best supported through the work-to-retirement transition. This scoping review summarises the literature on Australian farmers’ retirement experiences and the stressors they face during this transition. Barriers and facilitators that may hinder or help farmers were also explored. The relevant peer-reviewed literature was identified through database searching and the grey literature was collected via a web-based search. Seven studies were included in the review. Poor health and diminishing capacity to work was identified as a key stressor related to retirement. Other drivers of stress (i.e., pressure to live up to farming ideals, perceiving retirement as a threat to self-identity and financial concerns) overlapped with barriers to retirement. Farmers identified gradual transition, strong social networks, variety in interests and activities and early financial and succession planning as key facilitators of retirement. Findings will help inform the development of interventions to assist Australian farmers through this challenging stage of their lives.

## 1. Introduction

In Australia, retirement is loosely defined as the point at which an individual ceases employment, exits the workforce, or becomes eligible to access their primary income via the Government Age Pension or their superannuation [1,2]. It is a complex occupational transition that generally follows three general stages: retirement preparation, retirement transition and retirement itself [2,3,4]. The Agriculture, Forestry and Fishery industry has the highest average retirement age in Australia at 63.2 years (reference period 2018–2019 Australian financial year; [5]). Despite their average retirement age being 7.8 years above the national average, the reasons for delayed retirement in this industry, and what could be done to support workers through the work-to-retirement transition, has attracted little research. Seventy per cent of farmers and farm workers in the Agriculture, Forestry and Fishery industry work in crop growing and livestock farming [6]. Generational family farming remains the most common form of agricultural production in Australia [7], with the vast majority of Australian farms being owned and operated by families. More than half (55%) of agricultural workers in Australia are owners of a farming enterprise, and a further 26% are family members who also contribute to the farm (reference period 2016; [8]).

Farmers’ transition to retirement is heavily influenced by relationships between farming history, family, business, identity and place [9,10]. Family farming tends to be based on an ideal of generational ownership, with farm succession traditionally involving the transfer of physical farm work, farmer identity, status, control and ownership through the male line, ensuring family continuity with the land [11]. However, changes in the social, economic and environmental context over the past forty years have seen women take an increasing role in farm work and management [6], combined with a steady decline in generational farm succession practices overall [11,12,13]. Issues such as globalisation, new technologies, increasing climate variation, droughts and reducing water availability, as well as the declining numbers of younger farmers, have placed increased production and financial pressures on farmers [12,13,14,15,16,17]. Almost half of family farms in Australia are currently operated by a couple, without a younger generation to follow [8,18]. Many older farmers are delaying retirement and younger generations are being discouraged from pursuing farming careers [11,19,20]. Previous research has also highlighted that as retirement approaches, tensions often arise between traditional narratives of Australian family farming and farmers’ contemporary experiences [7,15,18].

Beyond ideals of generational ownership, the family farm exists as both a site of production and the family home, contributing to the construction of unique relationships between farmers and place [7]. Wythes and Lyons ([10]; p. 553) describe farmers’ identity as being “firmly linked with the land”, and this poses a unique set of challenges for farmers as they approach retirement. The emotional ties that farmers form with place are referred to as ‘place attachments’ [7], and help to maintain identity through ageing, offering stability, refuge and feelings of belonging during uncertain times and change [21]. For farmers, retirement may mean a severing of their connection with place (e.g., selling the farm and moving into town), and therefore may be experienced as a threat to their identity and sense of self. Often the farm has been passed down from previous generations. This is likely to contribute to many farmers experiencing feelings of loss and grief as they grapple with leaving the land [10]. The emotional fallout that farmers experience as they transition to retirement can be difficult for non-farmers to understand [15,20].

Some farmers may choose to remain on the farm in their older age and reduce their involvement with the more physically demanding tasks [22]. While this may maintain their connection with place and help to keep their sense of self intact, it can pose a new set of challenges. Farming is physically and psychologically hazardous work for farmers of any age, but older male farmers face particular risks to their physical and mental health [23]. Farmers aged over 55 years are more susceptible than younger ones to work-related injuries and machinery accidents [24,25], and experience health issues related to sustained physical labour, chronic stress, exposure to excessive noise, and long-term chemical use [24,26,27]. Moreover, ageing farming families experience increased social isolation, reduced quality of life, increased psychological distress and high rates of suicide compared to younger ones [15,23,28,29]. This is complicated by limited health and aged care services available in rural areas to support older farmers who choose to stay on the farm [30,31,32]. Moreover, the sustained presence of older generations on family farms, and delayed succession planning and financial handovers that often accompany it, can also pose challenges for younger generations who may lack freedom to make their own decisions (financial or otherwise) as a result [12].

Further compounding the challenges faced by older farmers considering retirement, are historical and cultural narratives of hard work and toughness, strength and stoicism and independence and pride that often inform farmers’ identities [33,34,35]. Many farmers derive their self-esteem, self-worth and respect within their communities from their work [15], which can shape negative attitudes and stigma towards retirement within farming communities. The perceived loss of value, meaning and respect from others through retirement can act as a source of shame [23], particularly for farmers who are unable to pass on the farm to younger generations [15].

For all these reasons, as highlighted by other scholars [10,15], there is a clear need to identify the most effective methods of supporting farmers as they move through this complex work-to-retirement transition. Therefore, the purpose of this research is to consolidate the literature on Australian farmers’ retirement experiences and the stressors they face when considering or preparing to transition to retirement. More specifically, we sought to identify barriers that may prevent Australian farmers from considering or planning for retirement early, as well as factors that may facilitate this transition to a new stage of their lives. Our focus is limited to Australia due to the contextual factors that are likely to influence this process, which we expect to vary between countries (e.g., pensions, superannuation). We anticipate that findings will help to inform development of contextually and culturally appropriate interventions to support farmers through the work-to-retirement process. This scoping review seeks to answer the following questions:

1. What stressors do Australian farmers face when considering or transitioning to retirement?
2. What barriers and facilitators may hinder or help Australian farmers during this transition?

## 2. Materials and Methods

This scoping review was conducted in accordance with the Joanna Briggs Institute (JBI) methodology for scoping reviews [36] and the Preferred Reporting Items for Systematic Reviews and Meta-Analyses extension for Scoping Reviews (PRISMA-ScR; [37]). The study protocol was registered in the Open Science Framework on 28 January 2022 (accessible here: https://doi.org/10.17605/OSF.IO/FVC5M).

### 2.1. Eligibility Criteria

Studies were included in the review if they explored Australian farmers’ experiences planning for or transitioning to retirement. Empirical studies using either quantitative or qualitative research methods and published in peer-reviewed journals were considered for inclusion in the review. The relevant web-based grey literature (e.g., reports prepared by government or non-government organisations) was also considered for inclusion. Included studies were limited to that written or published between 2001 and 2021 to ensure findings would be relevant to the current financial, environmental and global context. Opinion pieces and sources that were not published in English were excluded from the review.

The Australian Bureau of Statistics (ABS) combines farmers and farm managers when describing anyone who plans, organises, controls, coordinates and performs farming operations involving aquaculture, broadacre crops, horticultural crops and livestock [38]. Based on this definition, eligible studies explored the experiences of anyone who identified as a farmer or farm manager, or their family members who also live on a farm, given their known role in Australian family farming.

Studies exploring retirement among farm workers, labourers or casual employees who lived ‘off farm’ were excluded, as they were expected to have different experiences and levels of investment to farmers and farm managers. Studies exploring the experiences of those who retired into farming for lifestyle (non-commercial) purposes (e.g., hobby farmers) were also excluded because they were not considered to have the same blended business/financial and lifestyle demands as commercial-scale farmers who own and manage properties that have often been farmed by their family for generations. Studies that explored retirement from the perspective of successors were also excluded.

Studies were included in the review if most participants were aged 45 years or older, consistent with data collected by the ABS on retirement and retirement planning in the general population [5]. This allowed the perspectives and experiences of both farmers who had retired and those who were considering retirement to be captured. Studies that explored farmers’ opinions about retirement generally or hypothetical scenarios relating to retirement or succession planning were excluded from the review.

### 2.2. Search Strategy

An initial limited search of Cochrane Library, MEDLINE and Scopus was undertaken to identify key articles exploring retirement among Australian farmers. Key words in the titles and abstracts of relevant articles and index terms used to describe the articles were collated and used to develop a search string for MEDLINE (see Table S1). The search string was then adapted for each database search. Ten databases were searched between December 2021 and January 2022. Databases searched were Cochrane Library, the Cumulative Index to Nursing and Allied Health Literature (CINAHL), Embase, Emcare, JBI Evidence Synthesis, MEDLINE, PsycINFO, Rural and Remote Health Database (via Informit), Scopus and Web of Science. Forward and backward snowball searching was conducted to identify additional studies that cited or were cited by key journal articles that met the eligibility criteria. The grey literature was identified via a web-based search of Google using verbatim search mode, with the first five pages of results being considered for inclusion in the review [39].

### 2.3. Study Selection

**Figure 1:**
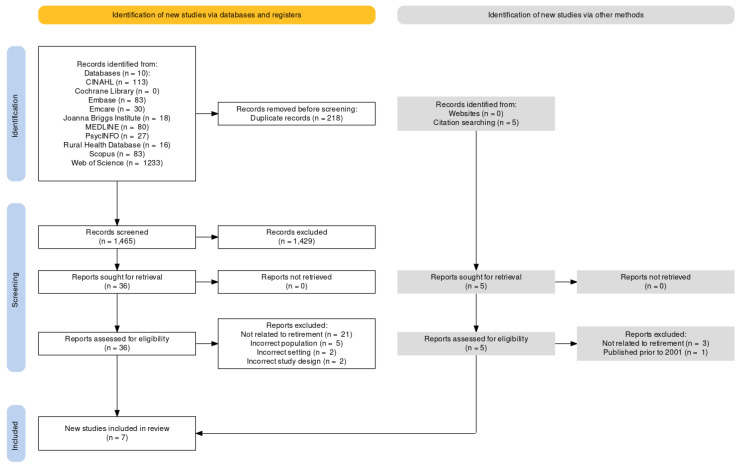
PRISMA-ScR flow diagram of the search strategy and study selection process. Note: PRISMA-ScR = Preferred Reporting Items for Systematic Reviews and Meta-analyses extension for scoping review; CINAHL = Cumulative Index to Nursing and Allied Health Literature.

Following the search, all identified citations were collated and uploaded into Covidence and duplicates were removed. Titles and abstracts were screened by two independent reviewers (L.S. and C.M.E.F.) and potentially relevant sources were retrieved in full. Full texts of potentially relevant sources were assessed in detail against the eligibility criteria by one reviewer (L.S.). Reasons for exclusion were recorded and are reported in Figure 1. Any disagreements between the reviewers (L.S. and C.M.E.F.) were resolved through discussion or with the senior author (K.M.G.).

### 2.4. Data Extraction

Data were extracted from included studies by one reviewer (L.S.) using a data extraction tool developed by the authors (see Table S2). The data extracted included specific details about the study aim, study design/methodology, sample size, participant characteristics, outcomes, key drivers of stress during the work-to-retirement transition, barriers to retirement and succession planning and facilitators of retirement and succession planning (see Table S3).

## 3. Results

### 3.1. Study Selection

The study selection process is presented in Figure 1. A total of 1698 studies were identified from the initial database and the grey literature search. Duplicates (*n* = 218) were then removed, and the remaining 1470 studies were screened via their title and abstract. Full texts were retrieved for 41 studies and assessed thoroughly against the eligibility criteria. Studies were excluded for the following reasons: they were not related to retirement (e.g., focused on health, wellbeing, or age-related issues more broadly or focused on financial or business-related aspects of farming); they were focused on an ineligible population (e.g., non-farming rural population or farmers planning to leave farming for reasons other than retirement); they were based in an ineligible setting (e.g., research conducted outside of Australia); they used an ineligible study design (e.g., narrative review); or they were published prior to 2001. Seven studies were identified and included in the review.

### 3.2. Study Characteristics

Table 1 summarises the characteristics of the seven included studies. The included qualitative studies used narrative [7,18], phenomenological [10], interpretivist [40] and ethnographic [34] approaches. Foskey [41] conducted a thorough review of the literature on retirement and farmers’ retirement experiences, followed by a mixed methods study to explore farmers’ retirement experiences in greater depth. From here, Foskey [41] developed, piloted and evaluated a retirement planning education program for farmers. The final study summarised a forum about ageing farmers that involved keynote presentations from three researchers and a panel discussion with policymakers and rural service providers [15].

The primary methods used for data collection were semi-structured interviews with farming couples [7,18], individual interviews [10,34,40,41], focus groups [40,41] and a discussion panel [15]. Most studies used a combination of collection methods and four studies also provided follow up data [7,18,34,41].

Four studies focused specifically on retirement and ageing within Australian farming and the issues that surround this experience [10,15,40,41], while another two studies explored how ageing and retirement influence farmers’ identity more broadly [18,34], particularly in terms of farm succession and attachment to place. A final study [7] extended previous research by the same authors [18], exploring how farming couples construct ‘generativity’, originally defined as concern for the wellbeing of younger generations [42], and how this influences retirement planning (e.g., by ensuring continuity with the land through generational farm succession).

### 3.3. Participant Characteristics

Perspectives of 101 participants (40–90 years old) were reported in a total of seven studies. Five studies included participants specifically from the Murray River regions of New South Wales (NSW) and Victoria [7,15,18,34,40], while two other studies included farmers from other areas of NSW [10,41]. Participants included active and retired farmers as well as people who had significant farming or other rural backgrounds (e.g., involvement in local council). In addition, Rogers [15] examined ageing farmers’ experiences of retirement from the perspective of the policymakers and service providers who worked with them, including financial counsellors, and aged care and mental health workers, who discussed how to better support ageing farmers during their transition to retirement.

### 3.4. Sources of Stress When Transitioning to Retirement

Significant overlap was observed between the sources of retirement-related stress and barriers to retirement, reported by farmers. For example, Foskey [41] described farmers perceiving retirement as an end to life and purpose, stemming from the intertwined relationships between home, occupation, identity and place. This contributed to farmers’ stress around retirement and planning for retirement, while also posing a barrier to them actually doing it. Similarly, farmers described feeling a sense of redundancy and identity loss when their valued interests and activities and relationships related to the farm were not well maintained through their transition to retirement [40]. Farmers preparing for retirement described a fear of not feeling valued or needed in their community [34]; a similar sentiment of not having anything meaningful to contribute beyond farming, was described by farmers who had fully retired [10]. Several studies [7,10,15,34] also described poor health and diminishing capacity to get work done as a key driver of stress around retirement. Poor health and diminishing capacity were the only sources of stress that did not also act as barriers to retirement. Other drivers of stress (e.g., fear of redundancy, fear of identity loss, financial concerns, having to sell the farm and lack of successor) were reflected in barriers to retirement identified, as detailed in Table 2.

**Figure 2:**
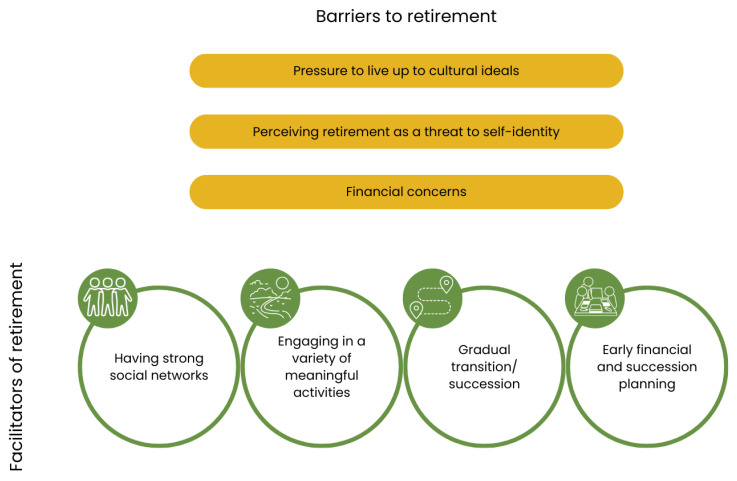
Barriers and facilitators faced by farmers when transitioning to retirement.

Figure 2 illustrates the barriers and facilitators that hinder or help farmers to transition to retirement. A summary of key themes is provided in Table 2 and Table 3, and each of these themes are described in detail below. Identified barriers to retirement were (1) pressure to live up to farming ideals (e.g., maintaining family farming continuity, maintaining independence, and being productive), (2) perceiving retirement as a threat to self-identity and (3) financial concerns (e.g., selling the farm, or burdening younger generations with debt) (Table 2). Identified facilitators of retirement were (1) having strong social networks, (2) engaging in a variety of interests and activities both within and outside of farming, (3) gradually transitioning to retirement or passing responsibilities onto successor and (4) early financial and succession planning (Table 3).

### 3.5. Barriers to Retirement

#### 3.5.1. Pressure to Live Up to Farming Ideals

Six studies identified that the expectation to live up to farming ideals posed a barrier to retirement. Subthemes within this barrier included pressure to keep the farm within their family [7,18,34]; to uphold their independence as a farmer [15,34,41]; to sustain productivity of the farm [15,34,41]; and to maintain masculine roles and stoic farming identities to be perceived as a ‘good farmer’ [18,34,41]. Downey et al. [18] acknowledged that differences in beliefs and ideals within couples may create tension because of their different perspectives towards retirement and potentially contribute to delays in retirement planning.

#### 3.5.2. Perceiving Retirement as a Threat to Self-Identity

Five studies reported that farmers perceived retirement as a threat to their identity and sense of self. Within this, farmers perceived retirement to threaten their social connections with their community and industry, leaving them fearful of social isolation [10,34]. Farmers described a sense of belonging that stemmed from being integrated and involved in their community [34]. When farmers were integrated into their community, they felt valued and needed [34]. Retirement threatened farmers’ sense of belonging, and thus their sense of value, through the perceived severing of their connection to their community [34]. This was especially heightened when farmers contemplated selling their farm and moving into town [15]. Perceptions of retirement being a threat to identity and sense of self were compounded by underdeveloped interests, activities and identities external to the farm [10,41]. Underdeveloped interests and activities outside of farming could furthermore contribute to retirement being perceived as a transition to ‘end of life’ [10,41]. This fear of idleness; of having nothing after retirement to provide them with a sense of meaning or to occupy their time, was likely to contribute to an avoidance of retirement planning or resistance to the lifestyle changes associated with retirement [7,15,34,41].

#### 3.5.3. Financial Concerns

Four studies highlighted how financial concerns could act as a barrier when transitioning to retirement with debt burden [7,18], with the potential need to sell the farm being an influential factor [15,18,41]. There was also a need for farmers to consider access to Government benefits such as the Age Pension as part of their decision making, which could contribute to frustration and delays in retirement planning [41].

### 3.6. Facilitators of Reitrement

#### 3.6.1. Having Strong Social Networks

Four studies reported that farmers who had strong social networks with family [10,18,41], the farming industry [41] and their wider community [10,34,41] were better supported through their to transition to retirement. In combination with sustaining meaningful existing relationships into retirement, it was also noted that farmers who developed new friendships in retirement found their retirement transition to be easier [10].

#### 3.6.2. Engaging in a Variety of Meaningful Interests and Activities

Four studies described having a variety of interests and activities outside of farming as an important facilitator for retirement. Allowing for continuity in farming interests and gradually exchanging these with meaningful alternative external interests could facilitate a smoother transition to retirement [10,40,41]. Farmers are in a unique position where they are often their own bosses, and their work is autonomous and flexible in nature [40]. This means that they can gradually adapt their work and workload so that they can sustain their involvement in farming in a way that is safe, well-defined and provides them with satisfaction as they transition to the next stage of their lives [40]. Programs and information aimed at supporting farmers through the transition to retirement should emphasise lifestyle planning and increasing engagement in non-farming-related interests and activities by reviving earlier interests and exploring new interests [41]. Lifestyle planning can help farmers to prepare for retirement by challenging negative perceptions and reframing retirement as a new stage of their lives that they can look forward to [41].

#### 3.6.3. Gradual Transition/Succession

Four studies identified that a gradual transition to retirement was an important facilitator [10,18,40,41]. Farmers reported feeling more content with their retirement journey if they were able to ease out of farming by altering their workload [10,40], moving in stages onto smaller properties [40] and presenting retirement as ‘downsizing’ rather than an abrupt exit [18].

#### 3.6.4. Early Financial and Succession Planning

Foskey [41] highlighted the importance of planning for retirement throughout the farming career. Discussions about future management of the farm should involve all family members, both on-farm and off-farm, to explore options and set up clear farm management roles and responsibilities. They emphasised that this should be an ongoing process that occurs over time [41].

## 4. Discussion

The purpose of this scoping review was to consolidate the literature on what is known about the stressors Australian farmers face when planning for or transitioning to retirement, with a focus on identifying barriers and facilitators that may hinder or help them through the work-to-retirement transition. Only seven studies met the criteria for inclusion; five used qualitative methods, one used mixed methods to explore farmers’ experiences of retirement and develop and evaluate a peer support program for farmers planning retirement, and the final study was a report on a forum presentation and panel discussion exploring farmers’ experiences of retirement. The most recent study was published in 2017, highlighting the need for further research in this field, given the multiple challenges faced by Australian farmers in recent years (e.g., drought, bushfires, COVID-19 epidemic, floods), and research that has suggested that retirement planning is particularly complex for farmers when they simultaneously face other challenges such as drought [43].

There was significant overlap between drivers of stress and barriers to retirement. Poor health and diminishing capacity to work was the only stressor identified that did not also pose a barrier to retirement. However, it is important to acknowledge how farmers’ declining health and capacity to perform physical labour interacts with their reported barriers to retirement. For example, pressure to live up to deeply entrenched ideals of the ‘good farmer’, who is hardworking, stoic and independent, conflicts with farmers’ experience of declining health as they age. Discourses throughout the literature characterise the ‘good farmer’ as having a strong work ethic involving working hard and long hours, keeping a tidy and productive farm that is recognised and garners respect from others in the farming community, and maintaining intergenerational continuity of the farm [15,34,35,44]. Findings from this review support the notion that this ideal is central to farmers’ identity, yet many ageing farmers struggle to uphold these expectations [15,34], particularly in the context of a changing social landscape and declining succession practices. When farmers are unable to maintain the good farmer identity, there is a sense of shame and failure [15,44], which may contribute to a reluctance to seek help or make decisions about retirement [34].

Similar cultural barriers are observed in research examining health-related help-seeking among farmers. Here, farmers display a tendency toward self-reliance, problem minimisation and normalisation which can prevent help-seeking for both physical and mental health problems [45,46,47,48]. This has implications for farmers’ health outcomes. For example, research has found that farmers who use behavioural disengagement (i.e., giving up or withdrawing effort from attempting to reach a goal with which a stressor is interfering) to cope with circumstances beyond their control experience higher levels of distress [49]. This is likely to also be true for farmers approaching retirement. Indeed, some research suggests that when longstanding historical and cultural ideals are threatened, farmers can experience poor self-esteem and self-worth, potentially contributing to suicidality [23]. This aligns with higher rates of suicide among male farmers over the age of 55 years [50] and should be considered when planning suicide prevention interventions for this at-risk group.

Findings from this review highlight farmers’ perceptions of retirement as a threat to their self-identity, stemming from place attachments, as well as underdeveloped interests and activities outside of farming, and neglected social networks which can lead to isolation in retirement. It is likely that the co-location of work and home, long work hours and strong connections to the ‘farmer identity’ contribute to a lack of diversity of interests and activities external to farming. When farmers retire without having developed interests or activities outside of farming, they may be left at ‘a loose end’ and with an abundance of free time. Some farmers describe a fear of ‘nothingness’ following retirement and perceive retirement as the end of life [10].

Consistent with this finding, Foskey [41] suggests while succession and financial planning for retirement are important, often there is an emphasis on these aspects and an underemphasis on broader lifestyle planning, which can leave farmers unprepared for life following retirement. Foskey [41] suggests that supporting farmers to gradually increase their non-farming-related interests and participate in new learning experiences allows for a smoother transition to retirement, while decreasing perceived threats to their identity and maintaining existing relationships within the farming community. Having developed interests and activities outside of farming helps to challenge farmers’ perceptions of retirement as ‘end of life’, instead reframing retirement as an opportunity—the beginning of a new life stage. In addition to broadening interests, based upon the findings from this review, it is recommended that lifestyle planning strategies help farmers foster social networks, as well as find ways to remain connected to place and their industry, in new (and likely lesser) ways as they enter retirement. Broader agricultural industry-wide campaigns to shift dominant farming cultural ideals that contribute to challenges to retirement (e.g., overly valuing productivity at the cost of wellbeing, independence, continuity of farm at all costs) may also help to address this issue. The need for these sorts of cultural shifts is something that previous scholars have also called for (e.g., Rogers et al. [15]). Shifting these social norms that are known to be detrimental to wellbeing is likely to be challenging, but would have multiple positive ramifications across the agricultural sector if achieved, such as improving productivity and helping to reduce suicide risk.

Notwithstanding the importance of lifestyle planning, findings from this review also highlight the continued need for financial and succession planning. Financial planning should occur throughout the farming career and, where able, family should be involved in farm management responsibilities from as early age as possible [41]. Early planning may help to prevent some of the interpersonal and financial challenges that are known to arise between family members when organising and preparing for farm succession [10,11,13,51]. To ease the transition to retirement, succession may occur gradually, through a process of ‘winding down’ workload [10,40], downsizing the farm over time [18,40], and/or handing over management to the next generation [40,41]. This gradual transition allows all parties to adjust to their new way of life, and for intergenerational learning to occur. There may also be a need to consider alternative farm and land management arrangements beyond traditional norms, such as co-operative farming and land trusts [15], particularly where there is no younger generation to take over the farm. Where farmers plan to maintain a role on the farm in retirement (or semi-retirement), it is important that this role is well-defined, to allow the younger generation to take on ownership and management responsibilities and facilitate exploration of their own farming and non-farming-related interests and activities.

As previously alluded to, it is clear from the literature included in this review, that farmers can be at risk of social isolation as they age [10,34], and that having strong social networks to support their transition to retirement is vital. Having a mix of social connections helps to increase farmers’ social support, while also giving them a new sense of purpose and value within their community during retirement. Farmers should be supported to maintain existing social connections associated with the agricultural industry, while also exploring new friendships in retirement. One way to facilitate this might be through programs designed to encourage farmers to ‘build a life beyond the farm gate’. Foskey [41] conducted a pilot program wherein farmers considering retirement were matched with a volunteer mentor who had already retired from the farming industry. The pilot program was successful in supporting farmers to prepare for retirement through financial planning, lifestyle planning and business/succession planning. One semi-retired farmer who participated in the program indicated that although he had been reluctant to withdraw completely from farm work, his attitude had changed, and he was becoming more active in non-farming-related activities within the broader community [41]. The research also highlighted the need for personalised retirement education that covers the full range of issues related to retirement planning (i.e., business/succession planning, financial planning and lifestyle planning) and challenges traditional cultural ideals that devalue life post-retirement [41]. Local agricultural shows and events may provide a useful avenue for offering this type of education to farmers. Being involved in agricultural shows may also provide retired farmers with a sense of purpose and value within their community, while also encouraging them to build new social connections.

Findings from this review are consistent with themes reported internationally [22,52,53,54,55], despite some differences in retirement and succession trends around the world [51]. Throughout the literature, references to farmers feeling ‘lost’ upon retirement [55], expressing a desire to remain ‘rooted in place’ [54] and continuing to play an active role on the farm [22,53], are present. Consistent with findings reported here, Riley [22] proposed that performing symbolic tasks on the farm allowed farmers in Hampshire and West Sussex in the UK to revisit the past and maintain their sense of worth and self-identity. Research among Swiss farming men and women similarly suggests that farmers’ identity is strongly linked to their work ethic [52]. Unlike in other countries, Swiss farmers tend to remain on the farm after retirement and continue to contribute to the farm business [52]. This allows them to uphold ideals of farm continuity within the family, maintain a strong work ethic and avoid perceived threats to self-identity posed by the severing of connections to place and work. Interestingly, Contzen et al. noted that the farmers who they interviewed did not demonstrate any desire for self-realisation beyond the family farm, stating “they do not plan to travel extensively or take up new hobbies as other retirees do, nor do they want to simply while away the time” ([52], p. 742). As described throughout this review, similar relationships exist between work, place, culture and identity for Australian farmers. Riley [55] described how underdeveloped interests and activities external to farming can disrupt the transition to retirement. Conversely, having flexibility in their everyday arrangements and activities (i.e., engaging in non-farming and off-farm activities) facilitated farmers’ adjustment [55]. In particular, women who performed multiple roles prior to retirement that necessitated social connections beyond the farm (e.g., motherhood, off-farm work) fared better than men whose occupational and social connections were deeply intertwined [55]. Researchers in Ireland have called for a national social organisation for older farmers to be established to help farmers to feel a sense of purpose, maintain industry connections and facilitate their transition to retirement [53]. Similar may be usefully implemented in the Australian context.

Findings from the review highlight a major limitation of this field of research in that most of the studies that have been carried out have focused on the experience of male farmers. Although farming has traditionally been a male-dominated industry, now 32% of agricultural workers in Australia are women (reference period 2016; [8]), and 49% of real farm income in Australia is produced by women (reference period 2006; [56]). Alston [57] suggests that women are the ‘new entrepreneurs’ of Australian agriculture, but it remains to be seen how the rise of female farmers will influence entrenched masculine farming roles and ideals around independence and stoicism, and what this will mean for attitudes towards retirement throughout the farming community. Traditionally, women around the world have been socialised to engage more with caring roles [58,59]. This tends to be something that women can continue to engage with into retirement (e.g., active involvement with grandchildren [18]), and it is something that women integrate into their identities (i.e., being a grandmother). This may mean that farming women may be less likely to perceive retirement as a threat to their self-identity or as ‘end of life’, as findings suggest may be the case for farming women in the UK [55]. However, further research is needed to examine Australian women’s experiences of retirement from (or on) the farm. Another significant limitation of this field of research is that all recent Australian research has explored the retirement experiences of crop and livestock farmers in New South Wales and Victoria. Given that Australia is a vast continent with a variety of geographical and climatic conditions, further research is needed to explore the retirement experiences of farmers within and between Australian states. Moreover, the Australian farming population is diverse [60], and in addition to crop and livestock farming, Australian farmers work across a range of other areas, including horticulture, viticulture, aquaculture and apiculture [8]. Further research may also examine differences in retirement experiences of farmers within and between sub-industries.

## 5. Conclusions

Moving from full time work to retirement is a particularly challenging life transition for farmers. New, creative, holistic efforts directed towards helping farmers with financial, succession and lifestyle planning, many years before they plan to retire, are warranted. Interventions of this type should include strategies to help farmers diversify their social networks, roles, interests and activities, while also finding ways to enable them to retain their sense of place, history and connection with the agricultural industry, to prevent problematic threats to their valued farming identities. Additional efforts towards creating broader cultural shifts of dominant farming cultural ideals that contribute to challenges to retirement (e.g., overly valuing productivity at the cost of wellbeing, independence, continuity of the family farm at all costs) are also likely to help mitigate challenges to farmers transitioning successfully to retirement.

## Figures and Tables

**Table 1:** Characteristics of included studies.

Author	Aim	Study Design	Methods	Sample Size	Participant Characteristics
Downey et. al. [7]	To examine the role of place identity in older farming couples’ retirement considerations.	Social constructivist narrative inquiry	60–90-min semi-structured interviews with couples	*N* = 6 couples	Aged 55–75 years Actively farming; farm sizes ranging from 505 to 2255 hectares Located in the Murray-Darling Basin in small rural district In a long-term relationship (between 20 and 50 years) At least one member of the relationship is a second-generation farmer
Downey et. al. [18]	To explore how older farming couples construct generativity throughout the life course.	Narrative study	60–90-min semi-structured, interviews with couples	*N* = 6 couples	Aged 55–75 years Actively farming; farm sizes ranging from 505 to 2255 hectares Located in the Murray-Darling Basin in a small rural district In a long-term relationship (between 20 and 50 years) At least one member of the relationship is a second-generation farmer
Foskey [41]	To understand how farmers define and experience retirement and ageing and the factors likely to influence their retirement actions.	Government report, involving literature review, mixed methods study and development, pilot and evaluation of peer support program	Literature review, individual semi-structured interviews, short survey, focus groups, follow-up focus group	*N* = 71 (service providers, *n* = 11; active and retired farmers, *n* = 60) *N* = 9 retired and semi-retired farmers participated in peer support program	Sample included 13 farming couples Aged 40–90 years Broad range of farming types, including crop, cattle, sheep and dairy North-Western and North coast of New South Wales (NSW)
O’Callaghan & Warburton [34]	To examine the impact of ageing and possible loss of the family farm on how farmers construct their situations and their self-identity.	Narrative ethnographic study	Individual interviews, observation, follow-up interview	*N* = 3	Male Born between 1946 and 1955 Currently living and working on family farm in Murray River region At least second-generation farmers Did not have children returning to family farm
Rogers et. al. [15]	To report on the outcomes of a policy and research forum on the demographic, economic, cultural, identity and health dimensions of ageing farmers.	Report on forum	Report on forum about ‘ageing farmers’, including summary of keynote presentations from three researchers and panel discussion with policymakers and rural service providers	-	Keynote speakers included three of the study authors (Barr, O’Callaghan and Brumby) Panel included representatives from Rural and Regional Policy (Department of Primary Industries), Rural Financial Counselling, Aged Persons Mental Health Service and Centrelink Forum attendees/participants included aged care and health sector workers, policy makers and rural service providers in NSW
Wiseman & Whiteford [40]	To report findings from a life history study exploring the retirement experiences of rural men.	Qualitative interpretivist study	Focus group, 60–90-min individual interviews	*N* = 8	Male Significant farming or other rural background (i.e., involvement in local council) Riverina region of NSW
Wythes & Lyons [10]	To explore the retirement experiences of rural men who have left the land.	Phenomenological study	60–90-min semi-structured, individual interviews	*N* = 7	Aged late 50s-late 60s All married and living with their spouse All fully retired (1.5–10 years) and had moved off the land Broad range of farming types, including crop, sheep, cattle, dairy, poultry, wheat and hay Most participants had a family history of farming and upon retirement from full-time farming had moved ‘into town’ with their spouse. Others had a gradual semi-retirement by scaling down the farm size before moving into a home in town.

**Table 2:** Key themes from the literature on farmers’ barriers to retirement.

Main Theme	Subtheme	Examples from Included Studies
Pressure to live up to farming ideals	Family farming continuity	Farmers express a sense of duty to the country and a desire to maintain generational succession and farming continuity within their family [7,18]. Challenges associated with ageing may be exacerbated when there is no younger generation to take over the farm [34]. Farmers may hold onto hope of family farming continuity even when their children have moved away and forged careers in other areas [34].
	Independence	Retirement is associated with ageing and can be interpreted as an acknowledgement that they are ‘old’ and therefore will lose their independence [41]. This goes against farmers’ need to be seen as independent, strong and capable by their community. There is a pressure for farmers to maintain independence, strength and capability regardless of age [15,34].
	Productivity	Farmers face pressure to live up to the ‘good farmer’ ideal by keeping active, busy, productive and maintaining the prosperity of the farm [15,34]. Farmers can feel shame when they are unable to physically keep up with the demands of the farm [41], reflecting on narratives of hard work to preserve their sense of self [34]. Farmers can struggle to seek help as this would acknowledge their diminishing capacity and increasing physical limitations [15].
	Rural masculinity and stoicism	Deeply entrenched cultural ideals and norms are upheld by stoic farming identity that idealises the good, strong, tough and hardworking farmer [34]. Farming couples are likely to have separate constructions and beliefs around retirement; conflict between ideals within the couple can contribute to delays in retirement decision-making [18]. Some farmers may find themselves at odds with their peers for considering retirement and “[taking their] knowledge and understanding out of the industry” [41].
Perceiving retirement as a threat to self-identity	Neglected social networks	Farmers can neglect their social lives as they age, causing them to become lonely and isolated. Older farmers feel isolated from other farmers but have limited energy to engage with them [34]. Farmers derive a sense of belonging from their community; when they are integrated and involved in their farming community, they feel valued and needed, but when this does not happen, they feel alone and isolated [34]. Lack of close social or family contacts can make it more difficult to cope with the experience of retirement and farmers can experience loneliness, especially when their friends have moved away or died [10]. Farmers who move into town to retire may feel ‘out of place’ among those who live in the town, perceiving that they have nothing in common, nothing to talk about and would not understand who they were or their life’s work [10].
	Connection to place and work	Farmers can perceive retirement as a negative, life-disruptive event that threatens their identity and self-worth, potentially leading to a resistance to change [7,15,34,41]. Farmers can perceive retirement as leaving their home, land and community [7,10,15]. Farmers talk about being ‘rooted in the land’, displaying a deep, embodied emotional attachment to place, which informs farming identity [34]. Co-location of work and home can cause emotional fallout when retirement means selling the farm and moving off the land [15]. Farmers are reluctant to leave a working life that has given them purpose and a sense of personal and social value [34].
	Underdeveloped interests and activities external to farming	Farmers often retire without developed interests or activities that are external to farming, leaving them at a loose end with their newfound free time [10]. For many farmers (but particularly male farmers) there is a strong association between the instrumental tasks of farming, the ‘doing’ and their sense of worth and self-identity [41]. Retirement can therefore be associated with a perception of worthlessness with no meaningful activities or tasks to fill the day. Emphasis on succession planning and financial planning can mean that other aspects of retirement, such as lifestyle planning, are overlooked [41].
	Connotations of retirement as end of life	Farmers may perceive retirement as ‘end of life’, e.g., “I know a lot of farmers that retired. They’d moved off the farm and into town and within 6 months they’d gone” [10]. Programs and information aimed at supporting farmers through the transition to retirement can tend to focus on succession planning, which may inadvertently reinforce associations between retirement and end of life [41].
Financial concerns	Debt burden	Farmers are concerned with burdening the younger generation with farm debt and delay retirement to avoid passing on financial concerns [7,18].
	Selling the farm	Some farmers may need to consider selling their farm as part of the transition to retirement [7,15]. This creates tension between aspects of identity, history and cultural ideals [15].
	Access to Government Age Pension	Farmers may need to consider eligibility for Government Age Pension as part of retirement planning. This can be complicated for farmers due to co-location of the family home and farm [41].

**Table 3:** Key themes from the literature on farmers’ facilitators of retirement.

Main Theme	Subtheme	Examples from Included Studies
Having strong social networks	Family networks	Connection with family may change throughout life course, but important to note maintaining connection with family networks supports smooth transition to retirement [41]. Farmers who have social contacts or family living nearby report enjoying retirement because they can be involved with grandparenting or helping with odd jobs [10]. Involvement with grandparenting offers opportunity for feelings of achievement and a way to express generativity (i.e., care for wellbeing of future generations) [18]. Farmers describe their wives as having a significant impact on their retirement decision-making and decline in health of wife may be a trigger for retirement for some farmers [10].
	Involvement in community	Being integrated and involved in the community can help farmers to feel a sense of belonging, and that they are valued and needed [34]. Involvement in off-farm activities in the community can help to broaden social networks [41]. Farmers with a range of interests and who are involved in diverse social networks report less stress transitioning to retirement [41]. Involvement in social groups as a retiree can help to develop sense of purpose and meaning in life [10].
	Industry networks	Some farmers may reduce their involvement in farm-related tasks as they prepare for retirement, while increasing their involvement in industry groups to maintain involvement in agriculture as they transition to retirement [41].
	New friendships in retirement	Retirement can provide an opportunity for broadening friendship networks and farmers can see this as a benefit of retirement [41]. Farmers describe necessity of friendships through transition to retirement, e.g., “You’ve got to sort of come out of your shell, otherwise you don’t make friends and friends are important. You can’t go on without them” [10].
Engaging in a variety of meaningful interests and activities	Continuity of farming interests and meaningful external interests and activities	Farmers who perceive retirement as a positive change tend to have a range of interests, be involved in diverse social networks, have supportive social networks and be future-oriented, lifelong participants in learning [41]. Successful transition to retirement requires increasing non-farming interests while winding down work life [41]. Farmers who report enjoying retirement are those who are actively involved in hobbies, interests and social groups [10]. Farmers express that it is important for hobbies and activities to be meaningful, rather than simply being ‘mundane time fillers’ [10]. Meaningful interests and activities can provide a link between pre- and post-retirement life and protect self-identity through the transition [40]. Farmers are often their own bosses, and their work is autonomous and flexible in nature [40]. This can facilitate gradual transition to retirement while staying connected with their working life and farming identity.
	Planning a lifestyle beyond farming	Retirement can provide an opportunity to revive earlier interests or explore new interests [41]. Some farmers may need support to assist them to draw on, expand and develop areas of their life external to farming. This can help to re-orient farmers’ self-identity so that their self-worth is less strongly tied to their work [41]. Programs and information aimed at supporting farmers through the transition to retirement should emphasise the need for lifestyle planning, alongside financial and succession planning [41]. Need for support services and systems to assist farmers to age successfully, in a way that gives them dignity, self-fulfilment and independence where possible [34].
Gradual transition/succession	Winding down personal farming tasks	Farmers suggest a ‘phased retirement process’, whereby farmers can ‘ease out of farming’ instead of transitioning straight from full-time farming to full-time retirement [10]. Gradually adapting daily activities can facilitate reconstruction of self-identity in the context of ‘doing’ over time (i.e., daily activities inform sense of self) [40].
	Downsizing the farm over time	Transition to retirement can be facilitated by reducing space gradually, e.g., moving from farm to a house with a big yard and then to a town house with a courtyard [40]. Retirement may be presented as downsizing the current farm property, rather than leaving the property or farming altogether [18].
	Handing over management to the next generation over time	Gradual transition to retirement by handing over farm management and responsibilities to family and moving to a smaller house on the family property [40]. Intergenerational succession should be a stepped process, involving gradual withdrawal from the farm by the senior generation as the younger generation takes on increasing responsibility [41].
Early financial and succession planning	Preparing for retirement throughout farming career	Retirement can be treated as an ‘event’ (connected with end of life) rather than a new life stage for which farmers need to prepare. Barriers to retirement can be challenged by encouraging future generations to perceive retirement as a normal and positive part of life that requires preparation throughout their farming careers [41]. Planning future management of the farm should be an ongoing process that occurs throughout the farming career [41].
	Involving family in retirement planning	Discussions about future management of the farm should involve all family members, both on-farm and off-farm [41].

## Data Availability

Data extraction table available in Supplementary Materials (Table S3).

## Supplementary Materials

The following supporting information can be downloaded at: https://www.mdpi.com/article/10.3390/ijerph20032588/s1, Table S1: MEDLINE search string; Table S2: Data extraction instrument; Table S3: Data extraction table.
