# Supplementary material for: Stressors, Barriers and Facilitators Faced by Australian Farmers When Transitioning to Retirement: A Scoping Review
Source: Int J Environ Res Public Health. 2023 Jan 31;20(3):2588. doi: 10.3390/ijerph20032588 (PMC9915984; doi:10.3390/ijerph20032588)
Supplement: Supplementary file 1 [file ijerph-20-02588-s001.zip › ijerph-2114929-supplementary.pdf]

Supplementary Materials

Table S1. MEDLINE search string.

1.	Farmers/
2.	Agriculture/
3.	Farms/
4.	(((agricultur* or farming or "farm land" or farm-land or farmland or station* or husbandry or cultivat* or agrarian or ranch) adj4 (worker* or employee* or farmer* or owner* or manager* or labour* or labor* or ranch* or "family member*")) or (farmer* or "farming family" or rancher* or cultivator* or farmworker* or farm-worker* or "farm worker*")).mp. [mp=title, abstract, original title, name of substance word, subject heading word, floating sub-heading word, keyword heading word, organism supplementary concept word, protocol supplementary concept word, rare disease supplementary concept word, unique identifier, synonyms]
5.	1 or 2 or 3 or 4
6.	Retirement/
7.	Aged/
8.	Middle Aged/
9.	Pensions/
10.	Life Change Events/
11.	"Aged, 80 and over"/
12.	(retire* or aged or aging or elder* or pension* or "middle aged" or middle-aged or "middle adulthood" or "independent living" or "aging in place" or "lifestyle modif*" or "life chang*" or "life-chang*" or "life chang* event" or "life-chang* event" or succession or transition* or semi-retire or ((reduce* adj2 workload) or "work load")).mp. [mp=title, abstract, original title, name of substance word, subject heading word, floating sub-heading word, keyword heading word, organism supplementary concept word, protocol supplementary concept word, rare disease supplementary concept word, unique identifier, synonyms]
13.	6 or 7 or 8 or 9 or 10 or 11 or 12
14.	Occupational Stress/
15.	Stress, Psychological/
16.	Burnout, Professional/
17.	Burnout, Psychological/
18.	Work-Life Balance/
19.	Stress, Physiological/
20.	Financial Stress/
21.	Psychological Distress/
22.	Mental Health/
23.	Mental Disorders/
24.	Adjustment Disorder/
25.	Depression/
26.	Depressive Disorder/
27.	Mental Fatigue/
28.	Mood Disorders/
29.	"Quality of Life"/
30.	Self Concept/
31.	Anxiety/
32.	Anxiety Disorders/

33.	((occupation* or job or "job related" or job-related or profession* or workplace or work-place or "work place" or career or "work life" or work-life or "work related" or work-related or social or life* or mental or psychological or emotion* or adjustment or environmental or mood or depress* or anxiet* or perceive* or affective) adj4 (stress* or burnout or "burn out" or burn-out or challeng* or pressure* or hardship* or distress* or disorder* or depress* or anxiet* or wellbeing or well-being or "well being" or change or confidence or fatigue or illness* or balance or health* or quality* or satisfaction or worr* or fatigue or concern* or conflict or "self esteem" or self-esteem)).mp. [mp=title, abstract, original title, name of substance word, subject heading word, floating sub-heading word, keyword heading word, organism supplementary concept word, protocol supplementary concept word, rare disease supplementary concept word, unique identifier, synonyms]
34.	14 or 15 or 16 or 17 or 18 or 19 or 20 or 21 or 22 or 23 or 24 or 25 or 26 or 27 or 28 or 29 or 30 or 31 or 32 or 33
35.	exp Australia/
36.	(Australia* or "New South Wales" or Victoria* or "South Australia*" or Tasmania* or Queensland* or "New South Welsh*" or "Western Australia*" or "Northern Territory" or "Australian Capital Territor*").mp. [mp=title, abstract, original title, name of substance word, subject heading word, floating sub-heading word, keyword heading word, organism supplementary concept word, protocol supplementary concept word, rare disease supplementary concept word, unique identifier, synonyms]
37.	35 or 36
38.	5 and 13 and 34 and 37

Table S2. Data extraction instrument.

Author + Published Year	
Title	
Aim of Study/Research Question	
Study Design/Methodology	
Sample Characteristics<br>- Inclusion Criteria<br>- Exclusion Criteria<br>- Method of Recruitment	
Intervention + Outcome Measures	
Outcome	
Key Drivers of Stress	
Identified Barriers and Facilitators to Retirement	
Identified Barriers and Facilitators to Succession Planning	

Table S3. Data extraction table.

Author/Year	Objective/Research Question	Methodology	Participants	Methods/Intervention	Outcome	Identified Key Drivers of Stress
Foskey [41]	The first aim was to provide a greater understanding of how Australian farmers from the north-western and north coast of NSW define and experience retirement and ageing and to understand the factors likely to influence their retirement actions.<br>The second aim was to develop, pilot and evaluate a retirement planning education program for farmers from these regions with appropriate government and community sector services consultation.	Report and mixed methods study	**Total n=71** people participated in interviews and focus groups (service providers, n=11; active and retired farmers, n=60)<br><br>**Total n=9** retired and semi-retired farmers participated in peer support program<br><br>**Sample Characteristics:**<br>• Aged between 40 - 90 years<br>• Broad range of farming types represented (including crop, cattle, sheep, and dairy)<br>• 13 farming couples<br><br>Recruited via local service providers, through farmers' organisations and media publicity (press and radio).	**Methods**<br>• Individual semi-structured interviews<br>• Short survey<br>• Focus groups<br>• Follow up focus group<br><br>The fieldwork included semi-structured interviews and a short survey with active and retired farmers.<br>After the interviews, gender-specific focus groups of active and retired farmers were conducted.<br>Focus groups were also held for service providers.<br>Follow-up focus groups were held in March 2001 to report back to participants the initial research findings and recommendations.	Participants in this research identified three groups within farming that could benefit from accessing improved learning opportunities to assist them in the retirement transition process:<br>• The farmer who has already retired and is having difficulty with the transition<br>• The older farmer to help them prepare for the transition into retirement<br>• Educating young farmers to start preparing for the transition into retirement early in their career and to perceive retirement as a normal life transition	• Difficulties when considering retirement due to the links between home, occupation, identity, and place.<br>• Retirement being perceived as an end to life and purpose.<br>• Farmers feeling worthless by not "doing".
Wiseman and Whiteford [40]	Aim to report findings from a life history study to explore the retirement experience of eight older rural men.	Qualitative interpretivist study	**Total n=8**<br><br>**Sample Characteristics:**<br>• Male<br>• From the Riverina Region of New South Wales, Australia<br>• Have significant farming or other	**Methods**<br>• Focus group<br>• 60-to-90-minute individual interviews<br><br>Each participant undertook between two and three interviews	Strategies that the farmers employed while navigating the retirement process over time were *gradual transition* and *maintaining connection*.<br><br>Gradual transition included:<br>• Tapering off by changing what is being done on the farm, giving the farmer a sense of control, and allowing for	• Experiencing/fearing a sense of redundancy and identity loss when valued occupations and relationships related to farming life are poorly maintained during the retirement transition

Author/Year	Objective/Research Question	Methodology	Participants	Methods/Intervention	Outcome	Identified Key Drivers of Stress
			rural background (for example, involvement in the local council)<br><br>Recruited through *Older Men: New Ideas* groups in Albury and Holbrook (NSW) and via colleagues of the primary researcher who identified potential participants.	depending on when saturation level of the data was achieved. Focus groups and interviews were tape recorded, transcribed verbatim and thematically analysed.<br><br>Narrative data was collected to understand the eight interviewees life history and the strategies they employed as they navigated the retirement process over time.	flexibility which supports continual connection with work while transitioning into the next stage of life – majorly assisted by the fact that farmers are their own boss<br>• Handing over the farm through gradual changes to aspects of occupational life including space, place, responsibilities, and routine (for example, moving from farm to smaller property nearby town to eventually a townhouse)<br><br>Maintaining connection included:<br>• Sustaining relationships between people, places and knowledge associated with farming and rural life to reaffirm and maintain identity and self-esteem continuity<br>• Continued participation in valued occupations, especially those relating to farm work to give a sense of purpose and competence	
Rogers, Barr [15]	Aims to report on the outcomes of a policy and research forum on the demographic, economic, cultural, identity and health dimensions of ageing farmers.	Forum presentation and discussion panel report	The target group for the forum were a range of stakeholders including people who work in the aged care and health sector, policy makers and rural service providers. Keynote speakers included the three authors Barr, O'Callaghan and Brumby.<br><br>The panel included representatives from Rural and Regional Policy	**Methods**<br>• Speaker presentations<br>• Discussion panel<br><br>The forum focused on issues surrounding retirement for ageing farmers and keynote speakers presented three different topics:<br>1. The demographic and economic drivers of structural ageing in	In the absence of a next generation takeover, retirement is not seen as an option for many, and many farmers choose to maintain their full-time farming status rather than sell the farm.<br><br>Most farms do not generate the median household income and hence, farm businesses that do not invest in future income growth become progressively less attractive career options for the younger generation.<br><br>Exiting the farm generally requires a land sale or lease to provide retirement income.	• Potentially having to sell the farm to afford retirement.<br>• Not having the next generation to provide support or succession<br>• Poor health and diminishing capacity to get work done<br>• The pioneering farmer identity being put under threat when the farm viability is threatened (resulting in poor self-esteem, seeing themselves

Author/Year	Objective/Research Question	Methodology	Participants	Methods/Intervention	Outcome	Identified Key Drivers of Stress
			(Department of Primary Industries), Rural Financial Counselling, Aged Persons Mental Health Service, and Centrelink.	the farm sector (Barr)<br>2. The cultural and identity issues underlying retirement choices of farmers (O'Callaghan)<br>3. The health and wellbeing implications of ageing on-farm (Brumby)<br><br>The key presentations were followed by a panel discussion with a focus on policy and practice responses to the series of challenges faced by ageing farmers.	There is emotional fallout and wide-ranging impacts to deal with when farmers consider selling the farm as the farm is a place where the farmer has lived, connected to community, raised stock, improved the land, and created both family and personal memories.<br><br>The self-identity of farmers is so deeply rooted in this historical, cultural, and physical construct and the narratives of hard work and toughness, resilience, independence, and pride, that it becomes problematic when this ideal is under threat. When their occupation and farm viability is threatened (due to poor health or 'cease of all work' retirement), the result can be poor self-esteem and self-worth creating health problems and far too often resulting in suicide.<br><br>Policies and services designed to assist ageing farmers make the transition must be consistent with farmers psychological, physical, and financial capacity to deal with change – including how to manage their identity as a farmer. There is an urgent need for a cultural shift within farming to recognise that there is life after farming.	as not being a 'good farmer')<br>• Loss of a spouse<br>• Loss of relationships as family and friends sell up and move away
O'Callaghan and Warburton [34]	Aims to examine the narratives of three male, baby-boom Australian farmers to tease out the impact of ageing and the possible loss of the family farm on the	Narrative ethnographic study	**Total n = 3**<br><br>**Sample Characteristics:**<br>• From the Murray River region (NSW and VIC)<br>• Male<br>• Born between 1946 and 1955	**Methods**<br>• Individual interviews<br>• Observation<br>• Follow-up interview<br><br>The research was conducted in several	The past has provided farmers with a narrative map of ageing, one that involves ageing-on-farm, surrounded by a younger generation. However, this map is highly problematic, based as it is on traditional gendered roles and outmoded scripts of behaviour and expectations that provide the core of farming identity and masculinities. It no longer provides contemporary farmers	• Threatened loss of identity, loneliness, isolation, and not having a next generation to hand the farm on to<br>• Pride in families' past becoming a burden for the farmer self as they

Author/Year	Objective/Research Question	Methodology	Participants	Methods/Intervention	Outcome	Identified Key Drivers of Stress
	ways that they construct their situations and their self-identity.		• At least second-generation farmers<br>• Currently running the family farm by living and working on the land<br>• Did not have children returning to farm their land<br><br>Recruited via snowball sampling from a previous larger study's reference group.	phases including initial interviews, followed by days of observation and finally follow-up interviews (in some cases up to ten months later). All interviews were conducted in the participants' home over three to four hours. The opening question of 'tell me about your life on the farm' meant that the focus of the initial interview was on the everyday life and experience of the participant.<br>Findings discuss how these three farmers construct a narrative map for their future as older farmers.	with a future direction or narrative for ageing well, leaving older farmers to struggle as they deal with a dramatically shifting environment, facing challenges such as climate-related adversity, the reducing numbers of farmers, particularly young farmers, and the overall worsening economic climate as they try to sell their properties. Each of these factors not only increases the pressures on baby-boom farmers, but also raises questions about the long-term sustainability of keeping ageing farmers on the land.<br><br>For all three farmers it was seen that a threatened loss of identity, loneliness, declining health, the farm slowly being neglected, being isolated from a world outside of farming, and not having a next generation to hand the farm on to, posed challenges as they age.	consider a life beyond farming<br>• Financial concerns<br>• Age-related physical challenges & watching the farm 'fall down around them'<br>• Fear of losing their male farming identity - difficult to distance themselves from the physical, emotional, and cognitive farmer identity and establish a new role identity<br>• Not feeling valued or needed in their farming community<br>• Not having a successor<br>• Health concerns related to ageing
Downey [7]	Aim is to examine the role of place identity in older farming couples' retirement considerations.	Social constructivist narrative study	**Total n = 6 couples**<br><br>**Sample Characteristics:**<br>• Actively farming<br>• Anglo-Australian<br>• Located in the Murry-Darling Basin within a small rural district (NSW)<br>• Farm sizes ranging from 505 to 2255 hectares<br>• Aged 55-75<br>• In a long-term relationship	**Methods**<br>• 60-to-90-minute semi-structured, joint interviews at two points in time over an 18-month period located in-situ on the couple's respective farms<br><br>Time-point one interviews concerned (1) family history on the farm, (2) the factors influencing couples'	Twigger-Ross and Uzzell's (1996) five place identity principles were used to report the findings.<br><br>Data showed that place is intertwined with identity, and that within the Australian family farming grand narrative context, retirement threatens farmers' values, causing tensions within relationships and in some cases, legitimating procrastination about retirement.<br><br>Findings suggest that self-efficacy and self-esteem may inform each other in a farming context, whereas place may facilitate or	• Debt<br>• Lack of successor<br>• Having to let go of a place which they 'live and breathe'<br>• Ageing threatening self-efficacy<br>• Poor health

Author/Year	Objective/Research Question	Methodology	Participants	Methods/Intervention	Outcome	Identified Key Drivers of Stress
			• Length of relationship between 20 and 50 years<br>• At least one member of the relationship is a second generational farmer<br><br>Recruited via first author's presentation of the study at a community social event, the distribution of advertising flyers via postal mail and snowball sampling.	retirement decision making, (3) decision making processes and (4) couples' future plans.<br><br>Time-point two interviews focused on (1) couples' reflections following initial interviews, (2) changes to couples' retirement plans, (3) who couples had spoken to about the future, as well as (4) couples' considerations of two decision-making scenarios; 'How do you decide when to have a holiday' and 'You are thinking about buying a new car, not a farm vehicle. How would you make that decision?'	impede agency, thus influencing feelings about retirement.	
Downey [18]	Aims to explore how older farming couples jointly construct generativity across the generations within a distinct farming culture.	Narrative study	**Total n = 6 couples**<br><br>**Sample Characteristics:**<br>• Actively farming<br>• Anglo-Australian<br>• Located in the Murry-Darling Basin within a small rural district (NSW)<br>• Farm sizes ranging from 505 to 2255 hectares<br>• Aged 55-75	**Methods**<br>• 60-to-90-minute semi-structured, joint interviews at two points in time over an 18-month period located in-situ on the couples' respective farms<br><br>Interview focused on (1) family history on the farm, (2) factors impacting on retirement		• Generative concern (who will take over the farm?)<br>• Desire to leave a legacy<br>• Passing on debt, leading to farmers postponing retirement<br>• Tension arising between couples due to individual constructions of retirement<br>• Being the last of several generations on the farm

Author/Year	Objective/Research Question	Methodology	Participants	Methods/Intervention	Outcome	Identified Key Drivers of Stress
			• In a long-term relationship<br>• Length of relationship between 20 and 50 years<br>• At least one member of the relationship is a second generational farmer<br><br>Recruited via mail	decision making, (3) decision making processes, and (4) couples' plans for the future.		
Wythes and Lyons [10]	Aimed to explore the retirement experiences of a small group of rural men who, in retirement, have left the land.	Phenomenological study	**Total (n = 7)**<br><br>**Sample Characteristics**<br>• Aged between late 50s to late 60s<br>• All married and living with their spouse<br>• 6/7 resided on the land they worked on prior to retirement, 1/7 worked on the land but lived in a country town prior to retirement<br>• Most participants had a family history of farming and upon retirement from full-time farming had moved 'into town' with their spouse. Others had a gradual semi-retirement by scaling down the farm size before	**Methods**<br>• 60-to-90-minute semi-structured, individual interviews<br><br>Participants were asked questions regarding enjoyment or satisfaction of working life and issues surrounding retirement (why, how, expectations, feelings about moving, changes, impacts and reflections with hindsight).	From the interviews, the following thematic themes were identified:<br>• Retiring from the land: significance of the land and the retirement transition<br>• Centrality of relationships: marriage partnership and social interactions<br>• Being 'involved' in life<br><br>Conclusion:<br>Farmers approaching retirement should be encouraged to plan not only for financial stability but also for maintaining purpose and balance in life through meaningful activities and relationships.	• Sense of loss at having to leave the farm<br>• Being idle, bored, dissatisfied, socially isolated and lonely<br>• Hearing stories of other farmers' bad retirement experiences (those who don't cope with the change)<br>• Physical work becoming harder as you age<br>• Not having anything meaningful to do outside of farming

Author/Year	Objective/Research Question	Methodology	Participants	Methods/Intervention	Outcome	Identified Key Drivers of Stress
			moving into a home in town<br>• Participants were involved in a variety of agriculture including crop, sheep, cattle, dairy, poultry, wheat and hay<br>• All were fully retired and had terminated association with the land<br>• Retired between 1.5 and 10 years<br><br>Recruited via snowball sampling from a member of the farming community who informed acquaintances who met the inclusion criteria.			
